# Dog Blood Type DEA 1 in Two Municipalities of Luanda Province of Angola (Sub-Saharan Africa)

**DOI:** 10.3390/vetsci11090449

**Published:** 2024-09-22

**Authors:** Ana C. Silvestre-Ferreira, Hugo Vilhena, Ana C. Oliveira, José R. Mendoza, Maria Garcia Aura, Josep Pastor

**Affiliations:** 1 Department of Veterinary Sciences, University of Trás-Os-Montes and Alto Douro (UTAD), 5000-801 Vila Real, Portugal; 2 Animal and Veterinary Research Centre (CECAV), University of Trás-Os-Montes and Alto Douro (UTAD), 5000-801 Vila Real, Portugal; hcrvilhena@hotmail.com; 3 Associate Laboratory for Animal and Veterinary Science—AL4AnimalS, 5000-801 Vila Real, Portugal; 4 Center for Investigation Vasco da Gama (CIVG), Department of Veterinary Sciences, Escola Universitária Vasco da Gama (EUVG), 3020-210 Coimbra, Portugal; 5 Onevetgroup University Veterinary Hospital of Coimbra (HVUC), 3020-210 Coimbra, Portugal; 6 Casa dos Animais Veterinary Clinic, Luanda, Angola; olivecris@hotmail.com (A.C.O.); joseriveromendoza@gmail.com (J.R.M.); rudysnel1992@hotmail.com (M.G.A.); 7 Department of Animal Medicine and Surgery, Universitat Autònoma de Barcelona (UAB), Bellaterra, 08193 Barcelona, Spain; josep.pastor@uab.cat

**Keywords:** Africa, Angola, alloimmunization, DEA 1, blood typing, blood group

## Abstract

**Simple Summary:**

Blood transfusions are lifesaving procedures. In dogs, because of the absence of naturally occurring alloantibodies, the risk of an acute hemolytic transfusion reaction at the first transfusion is negligible, but mismatched transfusions might produce alloimmunization. Therefore, it is important to blood type both donor and recipient dogs prior to blood transfusion. The prevalence of dog blood groups varies geographically and between breeds. The aim of this study was to determine the prevalence of DEA 1 in a canine population in Luanda province in Angola (Sub-Saharan Africa) and to assess the risk of alloimmunization after a mismatched blood transfusion. Of the 112 dogs tested (59 males and 53 females), 52.68% were DEA 1 positive and 47.32% DEA 1 negative. Female dogs presented a tendency to be DEA 1 positive and males DEA 1 negative (*p* = 0.0085). In a first-time mismatched blood transfusion, the calculated probability of a dog becoming sensitized was 24.9% and the probability of an acute hemolytic reaction following a second incompatible blood transfusion was 6.21%. These results differ from those of other African regions. In line with the results, blood typing is recommended prior to transfusion.

**Abstract:**

In dogs, the risk of an acute hemolytic transfusion reaction at the first transfusion is negligible; however, mismatched transfusions may produce alloimmunization. To avoid fatal acute hemolytic reactions in subsequent blood transfusions, it is important to recognize blood groups and to blood type both the donor and the recipient. Prevalence of dog blood groups varies geographically and between breeds. Our aim was to determine DEA 1 prevalence in a canine population in Luanda (Angola) and to assess alloimmunization risk after a mismatched blood transfusion. Blood samples were typed using an immunochromatographic strip technique. Of the 112 dogs tested (59 males; 53 females), 52.68% were DEA 1 positive and 47.32% DEA 1 negative. Females tended to be DEA 1 positive, and males DEA 1 negative (*p* = 0.0085). In a first-time mismatched blood transfusion, the calculated probability of a dog becoming sensitized was 24.9% and the probability of an acute hemolytic reaction following a second incompatible blood transfusion was 6.21%. DEA 1 prevalence obtained was similar to that reported worldwide, but differs from other African countries. The risk of alloimmunization and acute hemolytic transfusion reactions in mismatched blood transfusions is higher than that in other African regions. Blood typing is recommended prior to transfusion.

## 1. Introduction

Dog blood group systems are defined according to species-specific antigens located on the surface of erythrocyte cell membranes and are defined according to antigenic recognition. Each individual might express an antigen to a varying degree (positive blood type) or not express a specific antigen (negative blood type) [1,2]. From the different blood group systems reported in dogs, the Dog Erythrocyte Antigen (DEA) is the most studied, with the DEA 1, 3, 4, 5, 6, 7 and 8 blood types recognized internationally after classification with polyclonal alloantibodies obtained from previously transfused dogs or with specific monoclonal antibodies [1,3]. Since they are inherited as a complex autosomal dominant allelic system, dog erythrocytes might co-express any combination of these blood types on their surface [4,5]. Within the DEA blood group system, DEA 1 has a strong antigenicity and results in blood incompatibility reactions. Formerly, DEA 1 was proposed to have three subtypes: DEA 1.1, DEA 1.2 and DEA 1.3, but it was demonstrated that a single monoclonal antibody can recognize these antigens that are expressed from strong to weak positivity. This level of expression is genetically determined, and the expression pattern remains constant [5,6]. Subsequently, additional blood groups have been discovered, with Dal [7] and Kai [8] being the most recent and important. Although dogs do not appear to have naturally occurring alloantibodies, and the first blood transfusion might be safe, a mismatched transfusion of DEA 1 positive to DEA 1 negative dogs produces anti-DEA 1 antibodies that might develop fatal, acute hemolytic reactions in subsequent incompatible transfusions [9]. Nonetheless, there is still controversy about the clinical importance of the naturally occurring alloantibodies against DEA blood types other than DEA 1 and testing is not routinely available [1,4]. For these reasons the present study is centered only in the DEA blood group.

Canine blood groups are known to vary between breeds and geographically [10]. For the most part, the prevalence of DEA 1 in canines is around 60% [6]. When looking to purebred dogs, prevalences between 39.89% and 91.3% were found [11], and in mongrels it can vary from 42.8% in canine blood donors from Italy and Spain [12] to 91.3% in Brazil [13]. In Sub-Saharan Africa, DEA 1 prevalence can vary from 78% in Zimbabwe [14] to 47% in South Africa [15] and 39.89% in Nigeria [16]. Geographical variation of DEA 1 prevalence can be observed in Table 1.

Various commercial methods have been used for blood typing in dogs, and for this purpose an immunochromatographic strip technique using monoclonal antibodies was developed and validated [6]. The aim of this study was to determine the prevalence of DEA 1 blood type in a canine population from Luanda and Viana, two municipalities of Luanda province in Angola, a country in the Sub-Saharan region of Africa, and to compare it to those from other African countries. As a secondary aim, the risk of alloimmunization and blood transfusion reactions after an incompatible blood transfusion was also assessed.

## 2. Materials and Methods

For this study, 112 dogs that visited the Clínica Veterinária Casa dos Animais at Luanda and Viana were enrolled. Whole blood samples were collected in EDTA tubes from dogs that needed blood work on their diagnostic plan. No blood samples were collected on purpose for this study. Blood typing was only performed when there was enough blood leftover from routine testing. No dog had any known history of previous blood transfusion, and no pregnant females were included in the study. 

All owners gave informed written consent for the use of surplus blood samples. From each animal, data on gender, age, breed and origin were collected. Breed determination was based on owner report at first admission to the clinic. After blood collection, the samples were kept refrigerated at 4ºC and blood typing was performed on the same day as blood collection. 

For DEA 1 blood type determination, an immunochromatographic strip technique using a murine monoclonal antibody (Lab Test DEA 1, Alvedia, Limonest, France) was used following the manufacturer’s instructions. Briefly, first 3 drops of buffer and then 1 drop of blood are added to the card well. Afterwards, the membrane is inserted into the mixture and results are read in 2 to 5 min. The presence of a red line in front of the “DEA 1” arrow indicates a positive reaction. The proportion of dogs testing positive (+) and negative (−) for DEA 1 within each breed is set when at least 7 dogs were tested. A chi-square test was used to compare statistically significant differences between variables (location, sex and breed). Statistically significant differences were set at *p* value < 0.05. Graph Pad v.8.0.1 commercial statistical software was used. 

Based on the observed DEA 1 frequencies, the statistical probability of a dog becoming sensitized from a first-time mismatched transfusion was calculated by the formula: % DEA 1 negative × % DEA 1 positive/100. The probability of the same dog developing an acute hemolytic reaction after a second mismatched transfusion was calculated by the formula: % DEA 1 negative × % DEA 1 positive × % sensitization for the first transfusion/10,000 [13,20]. 

## 3. Results

Of the 112 animals tested, 52.68% (n = 59) were DEA 1 positive and 47.32% (n = 53) were DEA 1 negative. Table 2 presents the demographic characteristics of the population tested (Luanda and Viana) and blood typing results. Breeds represented by only one dog were grouped in the same category (American Staffordshire, Basset Hound, Boxer, Bullmastif, Bull Terrier, Golden Retriever, Siberian Husky, Swiss Shepherd Dog, Pug and Weimaraner). There were no statistically significant differences between the two locations for sex or DEA 1 groups (*p* = 0.376 and *p* = 0.325), however female animals tended to be DEA 1 positive and males DEA 1 negative (*p* = 0.0085). The calculated probability that a dog will become sensitized following a first-time mismatched blood transfusion was 24.9% and an approximate 6.21% probability of an acute hemolytic reaction following a second incompatible blood transfusion was also found.

## 4. Discussion

Dog blood groups are known to vary between breeds and geographically [10]. In our study, DEA 1 positive dogs showed a similar prevalence (52.68%) to that reported worldwide (50 to 65%), as previously summarized [12], but when compared to other African geographic areas there are some discrepancies: in Zimbabwe (Southeast Africa), 78% of dogs were DEA 1 positive [14]; in Nigeria (West Africa), 39.89% [16]; and in South Africa, 47% dogs were of the DEA 1 positive group [15]. 

To the best of our knowledge, this is the first description of dog blood types in Angola (Southern Africa). The breed distribution of tested animals was representative of veterinary medical care center attendees and the general population of the studied municipalities. Because defense dogs are popular in Angola, these were the most represented when considering the overall sample. There are no known native breeds to Angola, but the Boerboel, a large mastiff-type dog native to South Africa, was one of the most represented in our sample. Our results related to this breed are slightly different from those found by van der Merwe et al. (2016) in South Africa, which found a prevalence of 73% of DEA 1 positive Boerboel dogs, but more animals were tested in that study. Regarding German Shepard dogs, as in our study, a high prevalence of DEA 1 negative dogs (81.1%) was previously described [19]. The Rottweiler breed is documented with a 90% prevalence of animals which are DEA 1 positive [19,26], and in our study, the Rottweiler breed presented the highest DEA 1 positive prevalence (91.7%). Considering mixed breed dogs, they were the most represented in our sample and showed a similar prevalence (50%) of DEA 1 positivity as that previously described of 42.8 to 91.3% found in a combined study of 17 different countries [11], or 60% [6]. Regarding sex, our results are in accordance to those found in Zimbabwe, with females tending to have a higher expression of DEA 1 blood type [14]; however, other studies did not find a sex predisposition [18,26]. Females’ tendency to a higher expression of DEA 1 blood type may be related only to the sampling carried out in this study.

Knowledge of dog blood types after blood typing can prevent and minimize the risk of blood transfusion reactions and the induction of alloantibodies against RBCs because of blood incompatibilities. Therefore, it is of utmost importance to know the prevalence of blood types in diverse breeds and locations. For clinical purposes, DEA 1 negative dogs are considered the preferred blood donors [4]. In our study, the relatively low proportion of DEA 1 negative dogs makes the search for blood typed donors an important step in blood transfusion episodes. As to the probability of a recipient dog becoming sensitized following a first-time transfusion with blood that was not typed or cross-matched, as well as the probability of an acute hemolytic reaction following a second transfusion with blood from any other donor in the absence of pretransfusion compatibility testing, our results were similar to those found in Portugal [20]; although they were higher than those found in Turkey [27] or Zimbabwe [14], reinforcing the need for pretransfusion compatibility testing by blood typing and crossmatch. 

However, in dogs, although the DEA 1 antigen has been shown to be very antigenic and capable of eliciting a strong and long-lasting immune response, there is no evidence that alloimmunization occurs in 100% of incompatible transfusion episodes. Therefore, caution should be exercised regarding the likelihood of a dog in the examined population developing alloimmunization and experiencing an acute hemolytic transfusion reaction following an incompatible DEA 1 transfusion.

These results strongly support the idea that knowledge of breed blood types is helpful for building blood donor programs and recruiting donors during emergencies as well, especially if resources are limited for testing. 

New dog blood groups have been described. The Dal blood type was initially described based on the identification of an acquired alloantibody in a Dalmatian dog [7]. A high percentage of Dal negative Dalmatians and Doberman pinchers have been reported in North America [29] and Germany [17]. More recently, an investigation on the prevalence of two new blood groups, Kai 1 and Kai 2, produced by mouse hybridoma techniques found that most dogs in North America were Kai 1 positive/Kai 2 negative [8]. There is no proven relationship between Dal, Kai and DEA blood groups [8,12]. Because the clinical relevance of Dal and Kai blood types in transfusion medicine is still unknown and testing is not currently routinely available, the present study is centered only on DEA blood groups. Blood incompatibilities related to other blood groups than DEA 1 can only be evaluated by crossmatching [12]. This study has some limitations, mainly that it is a local study conducted in a low number of dogs, and that some breeds are poorly represented. Additional caution is warranted when interpreting the results, and outcomes should be carefully extrapolated to other geographic areas.

## 5. Conclusions

To the best of our knowledge, this is the first study on the prevalence of blood type DEA 1 in a canine population in Angola. In general, DEA 1 frequency was similar to that reported worldwide, but when compared to other Sub-Saharan African countries, some differences were found. The risks of alloimmunization and acute hemolytic transfusion reactions in mismatched blood transfusions were higher than in other African regions. DEA 1 blood typing before blood transfusion is recommended. Further studies should be carried out with a larger number of animals and in a larger geographical area.

## Figures and Tables

**Table 1:** Canine DEA 1 global distribution.

Continent/Country	Nº of Dogs Tested	DEA 1+ (%)
Africa		
Angola [this study]	112	52.7
Nigeria [16]	178	39.89
South Africa [15]	234	47
Zimbabwe [14]	100	78
Europe		
Germany [17]	206	59.2
Italy [18]	1037	62
Italy [19]	7414	61.2
Portugal [20]	274	56.9
Spain [21]	206	53.4
Spain [22]	92	75
Switzerland [23]	304	53
America		
Brazil [13]	150	91.3
NorthAmerica (Pennsylvania) [6]	66	69.7
(Pennsylvania) [24]	88	55
(Pennsylvania) [8]	503	59.6
(Pennsylvania) [25]	43	53.5
(California) [26]	6469	61.2
Asia		
Turkey [27]	178	65.2
Turkey [10]	198	61.1
India [28]	125	61.6

**Table 2:** Demographic characteristics and blood typing results of the 112 dogs tested.

	Nº of Dogs	DEA 1+ N (%)	DEA 1− N (%)
Gender			
Male	59	24 (40.68)	35 (59.32)
Female	53	35 (66.04)	18 (33.96)
Municipalities			
Luanda	84	42 (50)	42 (50)
Viana	28	17 (60.71)	11 (39.29)
Breed			
Mongrel	34	17 (50)	17 (50)
Boerboel	12	6 (50)	6 (50)
German Shepherd	12	3 (25)	9 (75)
Rottweiler	12	11 (91.7)	1 (8.3)
Pit bull	10	2 (20)	8 (80)
Poodle	7	6 (85.7)	1 (14.3)
American Bully	4	2	2
Labrador Retriever	4	4	
French Bulldog	3		3
Dogo Argentino	2	1	1
Pinscher	2	2	
Other	10	5	5
Total	112	59 (52.68)	53 (47.3)

## Data Availability

All the data supporting the results are included in the article. The dataset is available from the corresponding author on reasonable request.
